# Supplementary material for: EnzML: multi-label prediction of enzyme classes using InterPro signatures
Source: BMC Bioinformatics. 2012 Apr 25;13:61. doi: 10.1186/1471-2105-13-61 (PMC3483700; doi:10.1186/1471-2105-13-61)
Supplement: Addtional file 5 — The Java code to format the data files, evaluate and predict. The file enzml_java_code.tar.gz contains the Java code used to format database data to ARFF and XML formats, to execute cross and train-test (jackknife) evaluations and to record evaluation results to database. More information is included in the readme.txt file and the Javadoc files. The code can be used with a MySQL database. To use a different database software, other JDBC drivers might be required. [file 1471-2105-13-61-S5.gz › java_code/utils/doc/uk/ac/ed/inf/utils/setutils/class-use/Set.html]

Uses of Class uk.ac.ed.inf.utils.setutils.Set


---


|  |  |  |
| --- | --- | --- |
| **Overview** \| **Package** \| **Class** \| **Use** \| **Tree** \| **Deprecated** \| **Index** \| **Help** | |  |
| PREV   NEXT | **FRAMES**    **NO FRAMES**     **All Classes** |  |


---


## **Uses of Class uk.ac.ed.inf.utils.setutils.Set**

| Packages that use Set | |
| --- | --- |
| **test.setutils** |  |
| **uk.ac.ed.inf.utils.setutils** |  |

Uses of Set in test.setutils

| Methods in test.setutils that return Set | |
| --- | --- |
| `static Set` | `SetTest.getSet(java.lang.String id, java.util.TreeSet<java.lang.String> elements)` |
| `static Set` | `SetTest.getSetA()` |
| `static Set` | `SetTest.getSetAB()` |
| `static Set` | `SetTest.getSetABC()` |
| `static Set` | `SetTest.getSetAC()` |
| `static Set` | `SetTest.getSetAD()` |

Uses of Set in uk.ac.ed.inf.utils.setutils

| Methods in uk.ac.ed.inf.utils.setutils that return Set | |
| --- | --- |
| `Set` | `Set.clone()` |

| Methods in uk.ac.ed.inf.utils.setutils that return types with arguments of type Set | |
| --- | --- |
| `java.util.TreeSet<Set>` | `SupersetsManager.createSupersets()` |
| `java.util.TreeSet<Set>` | `SupersetsManager.getSets()` |
| `java.util.TreeSet<Set>` | `Set.getSubsets()` |
| `java.util.TreeSet<Set>` | `SupersetsManager.getSupersets()` |

| Methods in uk.ac.ed.inf.utils.setutils with parameters of type Set | |
| --- | --- |
| `void` | `Set.addSubset(Set newsetToAdd)` |
| `int` | `Set.compareTo(Set otherSet)` |
| `boolean` | `Set.contains(Set set)` |

Constructor parameters in uk.ac.ed.inf.utils.setutils with type arguments of type Set

`Set(java.lang.String id,
java.util.TreeSet<java.lang.String> elements,
SupersetsManager manager,
java.util.TreeSet<Set> subsets)`

---


|  |  |  |
| --- | --- | --- |
| **Overview** \| **Package** \| **Class** \| **Use** \| **Tree** \| **Deprecated** \| **Index** \| **Help** | |  |
| PREV   NEXT | **FRAMES**    **NO FRAMES**     **All Classes** |  |


---
